# Bacterial exposure risk to the endoscopist's face while performing endoscopy

**DOI:** 10.1002/deo2.209

**Published:** 2023-01-24

**Authors:** Kentaro Hoshi, Hidezumi Kikuchi, Koji Narita, Yukari Fukutoku, Taka Asari, Kuniaki Miyazawa, Yasuhisa Murai, Yohei Sawada, Tetsuya Tatsuta, Keisuke Hasui, Hiroto Hiraga, Daisuke Chinda, Tatsuya Mikami, Phawinee Subsomwong, Krisana Asano, Akio Nakane, Shinsaku Fukuda, Hirotake Sakuraba

**Affiliations:** ^1^ Department of Gastroenterology and Hematology Hirosaki University Graduate School of Medicine Aomori Japan; ^2^ Department of Community Medicine Hirosaki University Graduate School of Medicine Aomori Japan; ^3^ Department of Microbiology and Immunology Hirosaki University Graduate School of Medicine Aomori Japan; ^4^ Institute for Animal Experimentation Hirosaki University Graduate School of Medicine Aomori Japan; ^5^ Department of Preemptive Medicine Hirosaki University Graduate School of Medicine Aomori Japan; ^6^ Department of Biopolymer and Health Science Hirosaki University Graduate School of Medicine Aomori Japan

**Keywords:** bacteria, endoscopy, face, infection control, *Staphylococcus*

## Abstract

**Objectives:**

Gastrointestinal endoscopy increases the risk of bacterial exposure to endoscopists. However, before 2019, most endoscopists did not pay attention to microorganism transmission from patients. This study aimed to investigate the incidence of bacterial exposure to endoscopists’ faces during gastrointestinal endoscopic procedures using the bacterial culture method.

**Methods:**

This was a single‐centered, retrospective study including endoscopists who performed various gastrointestinal endoscopy procedures at the Division of Endoscopy, Hirosaki University Hospital between August 31 and October 6, 2020. Endoscopists wore surgical masks and affixed pre‐sterilized films over them. Following the gastrointestinal endoscopic procedures, attached microbes were collected from the endoscopists’ surface films using sterilized swabs. Collected microorganisms were cultured on tryptic soy agar and 5% sheep blood agar, and the incidence of bacterial exposure was determined by bacterial culture positivity. Cultured bacteria were identified by gram staining and 16S rRNA gene sequencing.

**Results:**

Bacterial culture positivity was 12.6%, and it was significantly higher in therapeutic than in diagnostic endoscopy. Notably, therapeutic endoscopy increased bacterial culture positivity in colonoscopy, but not in esophagogastroduodenoscopy. *Staphylococci*, including *Staphylococcus epidermidis* and *Staphylococcus capitis*, were the most commonly found bacteria in samples identified through 16S rRNA gene sequencing.

**Conclusions:**

The risk of bacterial exposure to the endoscopist's face was increased in colonoscopy treatment procedures. Therefore, endoscopists should be aware of the significant risk of microbial infection from scattering fluid that comes from the endoscopy's working channel.

## INTRODUCTION

Gastrointestinal (GI) endoscopy increases the risk of bacterial exposure to healthcare workers (HCWs).^1^, ^2^ The main sources of droplet or aerosol production during GI endoscopy are patients’ body fluids, which spread from their mouth or nose through burping, vomiting reflex, and coughing, and from their anus through feces and fart. Previous reports described the transmission of bacterial infection from patients to endoscopists.^3^ A study published in 1976, reported for the first time that the endoscopist was infected by a patient.^4^ Standard precautions, including face masks, gloves, and gowns were recommended by the American Society for Gastrointestinal Endoscopy.^5^, ^6^ However, most HCWs did not pay attention to microorganism transmission from patients before 2019.^7^

After 2020, the coronavirus disease 2019 (COVID‐19) pandemic reminded HCWs of the importance of infection control during GI endoscopy.^7^ Investigators showed the risk of viral infection caused by GI endoscopy.^2^, ^8^ In addition, several infection control devices were produced, especially for GI endoscopy.^2^, ^9^, ^10^ Currently strict precautionary measures are recommended by the American Society for Gastrointestinal Endoscopy during GI endoscopy.^11^ Therefore, during and after the COVID‐19 pandemic, infection control protocols and prevention from microorganism transmission have become a gold standard for HCWs. However, the real‐world risk of microorganism exposure to endoscopists during GI endoscopy has remained unclear. In 2019, the risk of bacterial exposure to the endoscopist's face during endoscopy was reported.^12^ However, in that study bacteria were collected from an unsterile area, and risk factors were not discussed.

To the best of our knowledge, this is the first study to identify bacteria collected from a sterile area during various types of GI endoscopy, to investigate the incidence and risk factors of bacterial exposure to the endoscopist's face in clinical practice. We identified these bacterial species through gram staining and 16S rRNA sequencing methods.

## METHODS

### GI endoscopy procedure

This single‐centered, retrospective study included endoscopists who performed various GI endoscopy procedures, such as esophagogastroduodenoscopy (EGD), colonoscopy (CS), and endoscopic retrograde cholangiopancreatography (ERCP) at the Division of Endoscopy, Hirosaki University Hospital, between August 31 and October 6, 2020. This study consisted of a survey of bacterial contamination in the endoscopy room and a retrospective analysis of clinical information. The surveyed GI endoscopies were consecutively performed in the endoscopy rooms (No. 1 or 2 out of No. 1–4), randomly allocated by a medical clerk.

**FIGURE 1:**
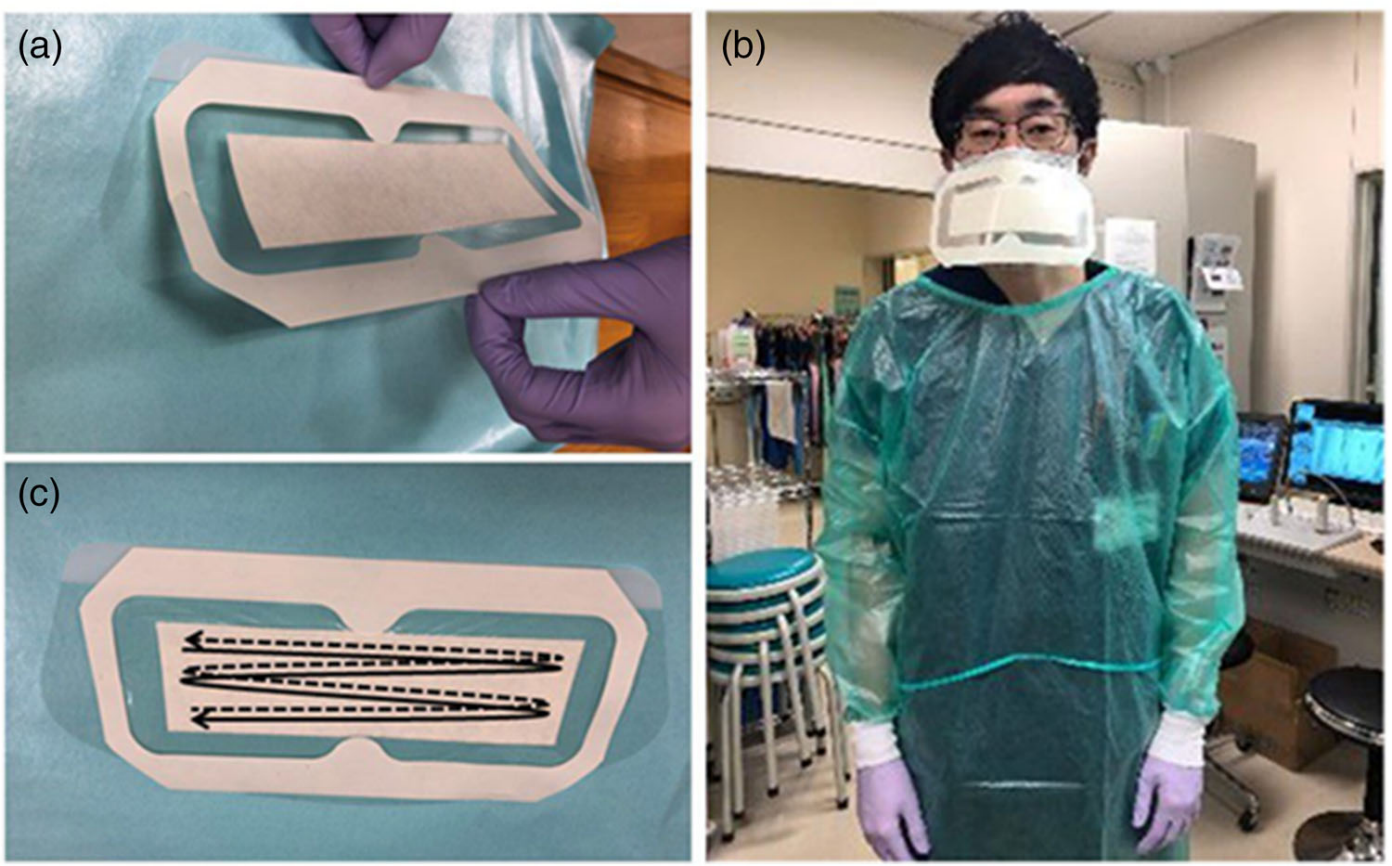
(a) A sterilized sheet was put in the center of the clear film. (b) The clear film with the sterilized area was affixed to the surgical mask. (c) Following the endoscopic procedure, the sterilized area was wiped off twice with a sterilized cotton swab.

During this period, patients did not use any infection control devices such as aerosol boxes or surgical masks. The endoscopists wore surgical masks with an affixed sterilized sheet. First, the sterilized sheet (Tegaderm+Pad Transparent Film Dressing, 3M Japan Limited, Tokyo, Japan) was placed in the center of the clear film (eye guard; 3M Japan Limited, Tokyo, Japan), and was then affixed on the surgical mask (Figure 1a,b).

**FIGURE 2:**
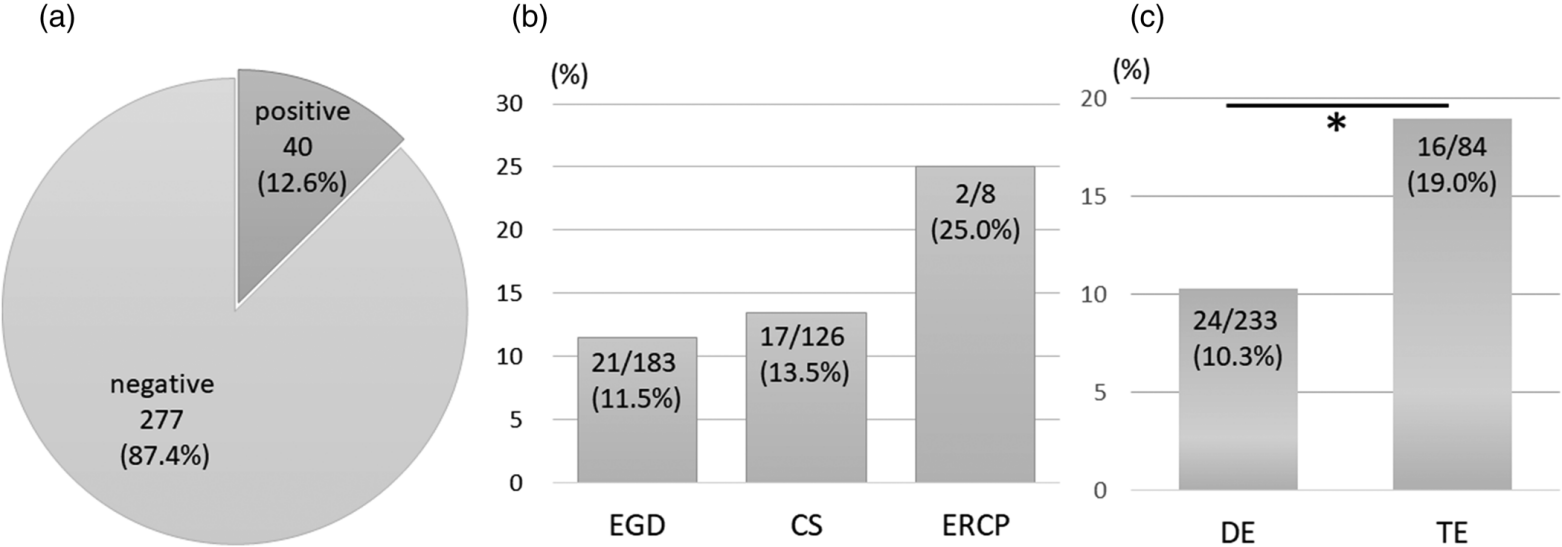
(a) Bacterial culture positive ratio in overall gastrointestinal endoscopy procedures. (b) Bacterial culture positivity by category of endoscopy. (c) Bacterial culture positivity by type of endoscopy, *p = 0.038.

This study was approved by the ethics committee of Hirosaki University School of Medicine (No.2022‐076) and conducted in accordance with the Declaration of Helsinki.

### Bacterial culture

Immediately following GI endoscopic procedures, the clear film was detached, and the sterilized area was wiped twice using a sterilized cotton swab (Pro‐media ST 25 PBS; ELMEX, Tokyo, Japan; Figure 1c). Samples were obtained by squeezing the cotton swab in 10 ml phosphate‐buffered saline followed by shaking for 60 s at 2000 rpm (EYELA CM‐1000; TOKYO RIKAKIKAI Co., Ltd., Tokyo, Japan) and centrifugation for 10 min at 3000 rpm (KUBOTA 5700; Tokyo, Japan). After discarding 9 ml of the supernatant, the remaining 1 ml was cultured on tryptic soy agar (BD Bioscience, Sparks, MD) and 5% sheep blood agar (Nissui Pharmaceutical Co., Tokyo, Japan) for 24 h at 37°C and bacterial growth were reported as colony‐forming positive or negative.

### Incidence and risk factors of the bacterial exposure

The incidence of bacterial exposure was determined by bacterial culture positivity. To evaluate bacterial exposure risk factors, patients’ clinical information (age and sex), category of GI endoscopy (EGD, CS, or ERCP), endoscopist's qualifications, examination time, and sedation used during endoscopy were retrospectively collected from endoscopy reports. This study categorized endoscopic procedures into two types: diagnostic endoscopy (DE) and therapeutic endoscopy (TE). DE included common diagnostic procedures, such as magnifying endoscopy, chromoendoscopy, digital image‐enhanced endoscopy, forceps biopsy, and endoscopic ultrasonography. Conversely, TE included several endoscopic therapies, including endoscopic balloon dilation, endoscopic mucosal resection, endoscopic submucosal dissection, and hemostasis. In addition, we compared bacterial culture positivity with or without a working channel procedure (WCP); WCP included not only TE but also biopsy, marking, and dye spraying. The working channel rubber plugs used in our hospital are reusable. Routinely, all rubber plugs were visually checked by a medical engineer to ensure they are intact, and most of them were used for about 4–6 months.

For sedation during endoscopy, benzodiazepines, pethidine, or dexmedetomidine hydrochloride were administered, as necessary. All endoscopists were classified into specialists (board‐certified fellows of the Japan gastroenterological endoscopy society) or non‐specialists. Vomiting reflexes during endoscopy and sliding hernia were evaluated in EGD cases. The vomiting reflex of patients during EGD was of two grades: none and mild, or severe. The presence of an esophageal sliding hernia was confirmed by an endoscopic image review.

### Identification of bacterial species

The cultured microorganisms were identified using gram staining and 16S rRNA sequencing methods (Macrogen Japan, Tokyo, Japan).

### Statistical analyses

All statistical analyses were performed using chi‐square tests with EZR (Saitama Medical Center, Jichi Medical University, Saitama, Japan),^13^ a graphical user interface for R (The R Foundation for Statistical Computing, Vienna, Austria), which is a modified version of R commander designed to add statistical functions frequently used in biostatistics. The threshold for significance was set at *p* < 0.05.

## RESULTS

### Clinical information (Table 1)

During the study period, a total of 317 endoscopic procedures EGD (*n* = 183), CS (*n* = 126), and ERCP (*n* = 8) were performed at our institution. There were 200 men and 117 women with a median age of 69 years. Endoscopies were performed by 21 endoscopists, 12 of whom were specialists, and 60/317 procedures were performed under sedation.

### The incidence of bacterial exposure in endoscopy (Figure 2)

Overall, bacterial culture from the sterile area in the mask was positive in 12.6% (40/317) of endoscopic procedures. The incidence of bacterial exposure during EGD, CS, and ERCP was 11.5%, 13.5%, and 25.0%, respectively. Notably, bacterial culture positivity during TE (19%) was significantly higher than that during DE (10.3%).

### Risk factors of bacterial exposure in EGD

We analyzed clinical information (Table 2), such as DE (*n* = 153) and TE (*n* = 30). DE included DE without WCP (*n* = 47) and DE with WCP (*n* = 106). TE included several techniques: endoscopic submucosal dissection (*n* = 8), endoscopic balloon dilation (*n* = 7), endoscopic hemostasis (*n* = 5), endoscopic injection sclerotherapy (*n* = 3), endoscopic mucosal resection (*n* = 2), ileus tubes insertion (*n* = 2), removal of foreign matter (*n* = 2), and esophageal radial incision and cutting (*n* = 1).

**TABLE 1:** Clinical information

Clinical information	*n*
Patient's median age, years (range)	69 (3–88)
Patient's sex, male/female	200/117
Category of endoscopy, EGD/CS/ERCP	183/126/8
Types of endoscopies, DE/TE	233/84
Endoscopists, specialist/non‐specialist	12/9
Sedation during endoscopy, without/with	257/60

**Abbreviations**: CS, colonoscopy; DE, diagnostic endoscopy; EGD, esophagogastroduodenoscopy; ERCP, endoscopic retrograde cholangiopancreatography; TE, therapeutic endoscopy.

**TABLE 2:** Incidence of bacterial exposure in esophagogastroduodenoscopy

Clinical information	Positive ratio	*p*‐Value
Age		
≥70 years	11.7% (12/103)	0.932
<70 years	11.3% (9/80)	
Sex		
Male	14.4% (18/125)	0.068
Female	5.2% (3/58)	
Vomiting reflex		
None or mild	12.0% (20/166)	0.447
Severe	5.9% (1/17)	
Sliding hernia		
Without	11.1% (19/171)	0.559
With	16.7% (2/12)	
Sedation during endoscopy		
Without	10.9% (16/147)	0.612
With	13.9% (5/36)	
Endoscopists		
Specialist	15.2% (16/105)	0.063
Non‐specialist	6.4% (5/78)	

**FIGURE 3:**
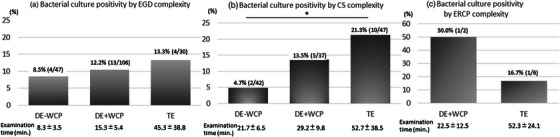
(a) Bacterial culture positivity and examination time by esophagogastroduodenoscopy (EGD) complexity. (b) Bacterial culture positivity and examination time by colonoscopy (CS) complexity. (c) Bacterial culture positivity and examination time by endoscopic retrograde cholangiopancreatography (ERCP) complexity. In CS, bacterial positivity of therapeutic endoscopy (TE) was significantly higher than that of DE (diagnostic endoscopy) without working channel procedure (WCP), *p = 0.030.

Bacterial culture positivity was 11.5% in all EGD procedures and was not significantly affected by patient characteristics, such as sex, age, with or without esophageal hernia, mild or severe vomiting reflex, and the type of endoscopists (specialists vs. non‐specialists). When compared among DE without WCP, DE with WCP, and TE, bacterial positivity was non‐significant at 8.5%, 12.2%, and 13.3%, respectively (Figure 3a). The examination time of DE without WCP, DE with WCP, and TE was 8.3 ± 3.5, 15.3 ± 5.4, and 45.3 ± 38.8 min, respectively.

### Risk factors of bacterial exposure in CS

CS was analyzed using various clinical information (Table 3), such as DE (*n* = 79) and TE (*n* = 47). DE included DE without WCP (*n* = 42) and DE with WCP (*n* = 37). TE included several techniques: colorectal polypectomy (*n* = 19), colorectal endoscopic mucosal resection (*n* = 18), colorectal endoscopic submucosal dissection (*n* = 6), and endoscopic hemostasis (*n* = 4).

**TABLE 3:** Incidence of bacterial exposure in colonoscopy

Clinical information	Positive ratio	*p*‐Value
Age		
≥70 years	14.5% (8/55)	0.761
<70 years	12.7% (9/71)	
Sex		
Male	11.6% (8/69)	0.493
Female	15.8% (9/57)	
Sedation during endoscopy		
Without	12.0% (13/108)	0.242
With	22.2% (4/18)	
Endoscopists		
Specialists	15.0% (12/80)	0.513
Non‐specialist	10.9% (5/46)	

**TABLE 4:** Incidence of bacterial exposure in endoscopic retrograde cholangiopancreatography

Clinical information	Positive ratio	*p*‐Value
Age		
≥70 years	28.6% (2/7)	0.540
<70 years	0% (0/1)	
Sex		
Male	33.3% (2/6)	0.345
Female	0% (0/2)	

Bacterial culture positivity was 13.4% in all CS, with significantly higher positivity in TE (21.3%) than in DE without WCP (4.7%; Figure 3b). In addition, WCP tended to increase bacterial culture positivity in DE (4.7% vs. 13.5%). The examination time of DE without WCP, DE with WCP, and TE was 21.7 ± 6.5, 29.2 ± 9.8, and 52.7 ± 38.5 min, respectively. Bacterial culture positivity was not significantly affected by patients’ sex and age, endoscopic background, such as with or without sedation, and endoscopists’ experience (specialists vs. non‐specialists).

### Risk factors of bacterial exposure in ERCP (Table 4)

In ERCP procedures, there were two cases of DE (cholangiopancreatography). All cases underwent ERCP under sedation. The bacterial culture positivity of DE with WCP and TE was 50.0%. and 16.7%, respectively (Figure 3c). The examination time of DE with WCP and TE was 22.5 ± 12.5 and 52.3 ± 24.1 min, respectively. Bacterial culture positivity was not significantly affected by sex and age.

### Identification of bacterial species (Table 5)

Twenty‐three colonies were found from 21 EGD cases, 19 colonies from 17 CS cases, and 2 colonies from 2 ERCP cases; these colonies were further evaluated. Microscopic evaluation revealed that 33 of 44 colonies were gram‐positive cocci.

Forty‐three bacterial samples were examined using 16S rRNA gene sequencing; we were unable to extract RNA from the remaining sample, which was obtained from CS. Identified bacterial species are shown in Table 5, distinguishing between with and without WCP. Bacteria from the genus *Staphylococcus*, including *S. epidermidis* and *S. capitis*, were predominantly identified in the tested samples.

**TABLE 5:** Identification of bacterial species

	EGD		CS		ERCP	
Bacterial species	**^−^** **WCP**	**^+^** **WCP**	**^−^** **WCP**	**^+^** **WCP**	**^+^** **WCP**	Total
*Staphylococcus epidermidis*	0	7	0	6	0	13
*Staphylococcus capitis*	0	1	3	3	0	7
*Micrococcus* sp.	3	0	0	0	0	3
*Enhydrobacter* sp.	0	2	0	1	0	3
*Uncultured staphylococcus*	0	2	0	0	1	3
*Bacillus flexus*	0	2	0	0	0	2
*Staphylococcus pettenkoferi*	0	1	0	1	0	2
*Brevibacterium frigoritolerans*	0	1	0	1	0	2
*Lysinibacillus xylanilyticus*	0	1	0	0	0	1
*Streptomyces* sp.	1	0	0	0	0	1
*Pantoea ananatis*	0	1	0	0	0	1
*Kocuria* sp.	0	1	0	0	0	1
*Staphylococcus aureus*	0	0	0	1	0	1
*Staphylococcus hemolyticus*	0	0	0	1	0	1
*Klebsiella pneumoniae*	0	0	0	1	0	1
*Microbacterium testaceum*	0	0	0	0	1	1

^−^WCP includes diagnostic endoscopy without WCP.

^+^WCP includes diagnostic endoscopy with WCP and therapeutic endoscopy.

Abbreviations: CS, colonoscopy; EGD, esophagogastroduodenoscopy; ERCP, endoscopic retrograde cholangiopancreatography; WCP, working channel procedure.

## DISCUSSION

To the best of our knowledge, this is the first report studying the incidence and risk factors of bacterial exposure to the endoscopist's face during different types of GI endoscopy. We identified cultured bacteria using 16S rRNA sequencing. Our data showed an overall risk of 12.6% for bacterial exposure to the endoscopist's face (40/317 procedures) in different types of GI endoscopy. These findings correlated with a previous report that described a 13.2% risk of mucocutaneous exposure from infectious body fluids to endoscopists.^14^ Although, most of the identified bacteria were nonpathogenic, endoscopists are exposed to microorganisms with a constant frequency.

In EGD, burping, vomiting reflex, and coughing are known mechanisms of bacterial scattering. We predicted that the incidence of bacterial scattering is high in patients who are young, have an esophageal hernia, or develop frequent vomiting reflex during EGD. Generally, the vomiting reflex is considered a risk of bacterial contamination. Herein, we demonstrated bacterial exposure risk from clinical records. Vomiting reflex severity had been recorded by endoscopists subjectively, and cases with severe vomiting reflex were few. However, there might have been individual differences among endoscopists in their judgment of vomiting reflex severity. Moreover, bacterial culture positivity did not differ between specialist and non‐specialist endoscopists. At our institution, non‐specialists have at least 3 years of endoscopy experience, and specialists mainly perform endoscopy in difficult cases, including DE with WCP and TE. Our results also showed that EGD with sedation tended to increase bacterial exposure. This could be because most EGD performed under sedation were complex procedures requiring longer examination time, or because patients who desired sedation tended to frequently burp or cough. Therefore, definitive risk factors of bacterial exposure in EGD could not be identified clearly in clinical practice. A previous report showed that diagnostic EGD did not lead to contamination of the endoscopist's face shield examined by the adenosine triphosphate method.^15^ However, this study also showed that bacterial culture positivity in EGD without WCP tended to be higher than in CS without WCP (8.5% vs. 4.8%), despite shorter examination times. Therefore, multiple factors besides examination time and WCP might be involved in EGD bacterial exposure. Conversely, bacterial exposure in CS might be more dependent on WCP and examination time. Scattering from the mouth is a theme warranting further research. Sampling swabs near the patient's mouth rather than the endoscopist's mask could increase bacterial culture positivity. Further, objective measurements are required to predict these risks more accurately. Moreover, vomiting reflex and coughing measurements that eliminate endoscopists’ bias are necessary.

In CS procedures, feces and farts from the anus are known to be major mechanisms of bacterial scattering. In CS, bacterial culture positivity increased in the order of DE without WCP, DE with WCP, and TE. Bacterial culture positivity in TE was significantly higher than that in DE without WCP. Examination time also increased in the same way. Therefore, bacterial contamination in CS might be more susceptible to WCP and examination time. Droplets on an endoscopist's face shield were increased during CS procedures in cases managed by less experienced endoscopists or with frequent WCP.^16^ Our study showed that the risk of bacterial exposure to non‐specialists in CS procedures was 10.9% (5/46 procedures), which was not higher than that of specialists (15.0%, 12/80 procedures). In our institution, most TE procedures were conducted by specialists. TE increases the risk of bacterial exposure, even when performed by specialists. This could be due to the frequent WCP. A previous report demonstrated that bacterial contamination is associated with damaged regions of working channels.^17^ During the COVID‐19 pandemic, Kikuchi et al. reported a new shielding method for endoscopic procedures, including working channels.^18^ The risk of bacterial exposure from working channels might decrease by using new infection control devices.

In ERCP procedures, the bacterial exposure risk to the endoscopist's face was the highest (2/8 procedure, 25.0%), albeit without significant difference. However, this may be due to the small number of ERCP procedures.

We identified bacteria that belonged to the genus *Staphylococcus* using 16S rRNA sequencing. *S. epidermidis* and *S. capitis* were frequently identified. Previous reports demonstrated that *S. epidermidis* and *S. capitis* were detected in the GI tract and from aerosols after dental procedures.^19^, ^20^, ^21^ *S. capitis*, mostly detected in CS, is a sepsis causative microorganism that occurs due to bacterial translocation from the intestine,^22^ causing severe infections, especially in children susceptible to infection. Furthermore, 16S rRNA gene sequencing in DE without WCP identified *Micrococcus* sp. (EGD, *n* = 3), S. capitis (CS, *n* = 3), and *Streptomyces* sp. (EGD, *n* = 1). Therefore, *Micrococcus* sp., a normal oral flora, might scatter from the mouth regardless of WCP. Various bacteria were identified in DE with WCP or TE, most of which were *S. epidermidis* (EGD, *n* = 7; CS, *n* = 6). Some of these may be exposed through WCP.

In our study, HCWs who performed the GI endoscopy were generally healthy, therefore the approximately 10% bacterial exposure may not be a clinical problem. Nevertheless, endoscopists should be aware of the risk of bacterial exposure because a case report showed an endoscopist who developed conjunctivitis after performing colonoscopy.^3^ This indicates that there is a risk of infection to the HCWs, depending on bacterial species or exposed site.

To examine bacterial contamination in the environment, we identified bacteria in the endoscopy room. From the analysis of 100 samples, bacterial colonies were detected in eight samples (8.0%), and the genus *Staphylococcus* was identified in three samples (3.0%) (*S. hominis*: n = 2, *S. epidermidis*: n = 1).

Before COVID‐19, most endoscopists did not pay attention to preventing bacterial infection because GI endoscopy is usually performed in a nonsterile environment. Specifically, during GI endoscopy endoscopists concentrate on the monitor; therefore, it is difficult to check the patient's surroundings and one's own body. The spread of COVID‐19 improved the focus on the importance of infection control measures for endoscopists. In terms of future infection control strategies, our findings support wearing facial protection, such as eye guards and surgical masks, as a standard precaution for all GI endoscopies. Endoscopists should also pay attention to working channel bacterial exposure. The working channel rubber plug should be disposable, whenever possible. When performing WCP, shielding the endoscope handle with a vinyl sheet, covering the device withdrawn from the working channel with gauze, and keeping the working channel away from HCWs may aid in effective infection control.

The limitations of this study include the single‐center nature and a small number of cases. Also, we were not able to collect all the patient's fluid. As microorganisms were cultured on tryptic soy agar and 5% sheep blood agar, we could only detect bacteria and no other microorganisms, such as fungi or viruses. In addition, it is difficult to determine whether the cultured bacteria were derived from the patients’ fluid. Most importantly, we did not directly probe whether the frequent use of a working channel during TE increased the risk of bacterial exposure. The actual frequency of device insertion/removal through the working channel was not recorded in this study. Therefore, further studies are required to address these concerns.

In conclusion, TE in CS increases bacterial exposure risk to the endoscopist's face. During GI endoscopy, endoscopists should be aware that not only direct patient's fluid but also scattered fluid from around the working channel is associated with a significant risk of infection. Unknown microorganisms might cause infectious diseases. Therefore, it is important to implement infection control procedures in the future.

## CONFLICT OF INTEREST

None.
